# Announcement

**Published:** 1895-07

**Authors:** 


### Editorial.

During the past twenty-six years of the present management of the SOUTHERN MEDICAL RECORD, the object has been to render it useful to the medical profession. In the original contributions, valuable papers have appeared in each number and in the selections, the most important subjects of a practical and scientific nature have been presented. Merely personal issues have been excluded from its pages so far as practicable, and the interests of no clique has been advanced by it, nor have the private interests of individuals been consulted in its management. There has been a constant effort to maintain a high standard for the character of the articles published, and it is believed that its contents have compared favorably with the matter contained in other monthly journals of the South.

The large number of medical journals issued monthly and weekly, with a single daily publication, indicates an increasing demand for medical knowledge among the members of the profession. While the readers are of great variety as to taste and mental aptitude in regard to the information demanded, there must be a practical bearing of every thing which constitutes the make-up of a medical journal. Viewing the mission of the SOUTHERN MEDICAL RECORD as that of fitting practitioners better for their daily service of healing the sick and curing their maimed and injured patients, it has been a prime consideration to afford the best literature of a practical nature for our readers. Mere themes or speculations have had a very small space in our pages, and it is held, beyond any question, that those who have diligently read each number have become more completely equipped for their professional work. It is, therefore, claimed that a good work has been accomplished and that it will continue under the new regime.

In transferring the RECORD to another, it is confidently expected that the character and standing of the work will be sustained, and even improved upon, so that the patronage may be increased. In the endorsement of the new administration, it is proper to state that for the most part, the same collaborators and contributors will render their co-operation in maintaining the status of the RECORD, and it is most cheerfully commended to the confidence and support of the medical profession.

In taking leave of the readers, who have so generously extended their forbearance to any shortcomings which may have occurred in the past, it is a source of satisfaction to feel that our kindly relations have not been marred by any asperity or bitterness, and that the separation will not be accompanied by any heart burnings. At least, it may be stated that upon my part, the retirement from this field of service, leaves only regret at the interruption of the pleasant relations existing with the patrons of the RECORD.

